# YKL-40 - an emerging biomarker in cardiovascular disease and diabetes

**DOI:** 10.1186/1475-2840-8-61

**Published:** 2009-11-23

**Authors:** Camilla N Rathcke, Henrik Vestergaard

**Affiliations:** 1 Dept of Internal Medicine, Center of Endocrinology and Metabolism, Copenhagen University Hospital Herlev, Denmark; 2 Faculty of Health Sciences, University of Copenhagen, Denmark

## Abstract

Several inflammatory cytokines are involved in vascular inflammation resulting in endothelial dysfunction which is the earliest event in the atherosclerotic process leading to manifest cardiovascular disease. YKL-40 is an inflammatory glycoprotein involved in endothelial dysfunction by promoting chemotaxis, cell attachment and migration, reorganization and tissue remodelling as a response to endothelial damage. YKL-40 protein expression is seen in macrophages and smooth muscle cells in atherosclerotic plaques with the highest expression seen in macrophages in the early lesion of atherosclerosis. Several studies demonstrate, that elevated serum YKL-levels are independently associated with the presence and extent of coronary artery disease and even higher YKL-40 levels are documented in patients with myocardial infarction. Moreover, elevated serum YKL-40 levels have also been found to be associated with all-cause as well as cardiovascular mortality. Finally, YKL-40 levels are elevated both in patients with type 1 and type 2 diabetes, known to be at high risk for the development of cardiovascular diseases, when compared to non-diabetic persons. A positive association between elevated circulating YKL-40 levels and increasing levels of albuminuria have been described in patients with type 1 diabetes indicating a role of YKL-40 in the progressing vascular damage resulting in microvascular disease.

This review describes the present knowledge about YKL-40 and discusses its relation to endothelial dysfunction, atherosclerosis, cardiovascular disease and diabetes and look ahead on future perspectives of YKL-40 research.

## Introduction

Since the results of the Framingham Heart Study revealed C-reactive protein (CRP) as a cardiovascular marker even in ranges considered normal [1-3], several studies of biomarkers in cardiovascular disease (CVD) have been conducted. Until this day, CRP remains the most validated biomarker but substantial knowledge about CRP as a predictor of cardiovascular events is now complemented by studies of new emerging markers such as interleukin 18, matrix metalloproteinase 9, adiponectin and CD40 ligand [4]. The present review focuses on the inflammatory protein YKL-40 and its role in atherosclerosis, CVD and diabetes.

### YKL-40 - biology and physiology in general

YKL-40 is a 40 kDa heparin- and chitin-binding glycoprotein also known as human cartilage glycoprotein 39 (HC-gp39) [5], 38-kDa heparin-binding glycoprotein [6] or chitinase-3-like protein 1 (CHI3L1) [7]. The abbreviation YKL-40 is based on the one letter code for the first three N-terminal amino acids, tyrosine (Y), lysine (K) and leucine (L) and the apparent molecular weight of YKL-40 [8].

The *CHI3L1 *gene for human YKL-40 is localized in a highly conserved area on chromosome 1q31-q32 [9] and the crystal structure of YKL-40 has been described [10]. YKL-40 belongs to the family 18 of glycosyl hydrolases comprising chitinases from various species [11], but YKL-40 is without any enzymatic properties [5,12,13].

YKL-40 is secreted *in vitro *by a variety of cells and seems especially involved in activation of the innate immune system and in cell processes in relation to extracellular matrix remodelling [11,14]. YKL-40 induce the maturation of monocytes to macrophages, and is secreted by macrophages during late stages of differentiation and by activated macrophages [7,15-18]. Studies show that the differentiation and maturation of CD14+ monocytes to CD14-, CD16+ macrophages are attended by an expression of YKL-40 from CD16+ macrophages [17]. YKL-40 has also been shown to be an adhesion and migration factor for vascular cells and is secernated by differentiated vascular smooth muscle cells (VSMCs) [6,19,20]. *In vivo *YKL-40 protein expression is found in human VSMCs in adventitial vessels [21] and in subpopulations of macrophages and VSMCs in different tissues with inflammation and extracellular matrix remodelling as in atherosclerotic plaques [14,19,22].

The knowledge about the physiological function and the mechanisms by which YKL-40 mediates its effects is still scarce. Immunohistochemical studies of different types of normal human tissues show, that cells with a high cellular activity, e.g. a high level of metabolic activity and/or proliferation, have especially high YKL-40 expression [23,24].

YKL-40 mRNA and protein expression are found in tissues from all germ layers and are present during the early development of the human musculoskeletal system where they seem associated with cell proliferation, differentiation and tissue morphogenesis [23]. Other studies show, that YKL-40 stimulates the proliferation of human connective tissue cells (fibroblasts, chondrocytes, synovial cells) in a dose-dependent manner in a functional concentration range similar to that of insulin-like growth factor (IGF-1). When present in suboptimal concentrations, YKL-40 and IGF-1 work in a synergistic fashion [25,26]. In mouse studies, YKL-40 stimulates the antigen-induced T-helper 2-response and seems to induce tissue inflammation and fibrosis mediated by IL-13. In this sense, YKL-40 plays an essential role in antigen sensitization and IgE induction as well as in activation of innate immune cells [27].

In fibroblasts and synovial cells YKL-40 mediates a mitogenic effect through initiation of mitogen-activated protein kinase (MAPK) and phosphoinoside-3 kinase (PI3K) signalling pathways by phosphorylation of the extracellular signal-regulated kinase-1 and 2 (ERK1/ERK2) and protein kinase B (AKT), respectively. Both pathways are required for the cells to complete mitosis and the activation of these pathways stimulates the growth of connective tissue cells [26].

In fibroblasts and chondrocytes YKL-40 reduces the activation of p38 and SAPK/JNK MAPKs which counteracts the inflammatory responses to TNFα and IL-1. This leads to reduced concentrations of matrix metalloproteinases (MMPs) and IL-8. The modulation of p38 and SAPK/JNK by YKL-40 is mediated through the PI3K [28] and the induction and continued secretion of YKL-40 require sustained activation of Nf-κB [29]. YKL-40 has no effect on the signalling pathways p38 and SAPK/JNK MAPKs when present without the presence of TNFα and IL-1 and similar do not affect the MMP or IL-8 production. This suggests that YKL-40 expression is an anti-inflammatory counteract of the inflammatory response mediated by TNFα and IL-1 [28] beside its apparent function as a growth factor [26]. The activation of cytoplasmatic signal-transduction pathways suggests, that YKL-40 interacts with one or several signalling components on the plasma membrane. However, specific cell surface receptors or potential YKL-40 ligands remain to be determined.

No difference in serum or plasma YKL-40 levels has been found between genders [11]. In serum, no significant diurnal, weekly or long-time variation in serum YKL-40 concentrations are found in healthy subjects [30]. Similarly, serum YKL-40 concentrations are not affected by physical exercise [30]. There seems to be no or only weak correlation between YKL-40 and hsCRP in studies of patients with diabetes, obesity or atrial fibrillation [31-34] whereas a positive correlation is found between YKL-40 and hsCRP in studies of patients with manifest coronary artery disease (CAD) [35,36]. Opposite CRP which is a systemic inflammation marker primarily secreted by hepatocytes in response to proinflammatory mediators such as IL-6, YKL-40 is locally produced and secernated. However, all studies investigating the association between YKL-40 and IL-6 found a positive correlation between the two [34,37,38]. Furthermore, a tight association between monocyte chemoatractant protein-1 (MCP-1) and YKL-40 have been found in morbidly obese patients [33]. MCP-1 is associated with monocyte trafficking and macrophage infiltration in adipose tissue [39] and it is also a strong predictor of cardiovascular death [40]. YKL-40 levels are elevated in morbidly obese patients, but despite the apparent linkage between YKL-40 and macrophage maturation and activation, no studies have ever found and association between YKL-40 and body mass index [31,33,37].

### YKL-40 in endothelial dysfunction and atherosclerosis

The participation of YKL-40 in inflammatory states and vascular processes implies that YKL-40 may play a role in endothelial dysfunction and atherosclerosis. In endothelial dysfunction, elevated YKL-40 levels seem to be involved in relation to cell migration, reorganization and tissue remodelling as a response to endothelial damage [6,20,41].

*In vitro *VSMCs from explants of swine thoracic aorta syntesize YKL-40 during the time of transition from monolayer culture to a non-proliferating differentiated multilayer culture [41,42]. The YKL-40 secretion continues during the reorganisation of the cells where multicellular nodules are formed. In these nodules the cells re-express markers of differentiated VSMCs [6,20,41]. This *in vitro *nodule forming process mimics some of the characteristics of the *in vivo *changes that occur in VSMCs following injury, where media smooth muscle cells dedifferentiate, migrate and contribute to the process of restenosis and neointima formation [43].

*In vitro *studies also show that YKL-40 promotes chemotaxis, cell attachment, spreading and migration of vascular endothelial cells which suggest a role of YKL-40 in the atherosclerotic plaque formation, where smooth muscle cells are induced to migrate through the intima in response to exogenous signals [20]. YKL-40 also modulates vascular endothelial cell morphology by promoting the formation of branching tubules, indicating that YKL-40 has a role in angiogenesis by stimulating the migration and reorganization of VSMCs [20]. These *in vitro *studies are supported by immunohistochemical analysis which has shown *in vivo *protein expression of YKL-40 in human smooth muscle cells in atherosclerotic plaques [19].

YKL-40 mRNA expression is highly up-regulated in distinct subsets of macrophages in the atherosclerotic plaque, a plaque that is characterized by the infiltration of monocytes into the subendothelial space of the vessel wall and a subsequent lipid accumulation in the activated macrophages. Particularly macrophages that infiltrate deeper in the lesion show high YKL-40 mRNA expression and the highest expression is seen in macrophages in the early lesion of atherosclerosis [22]. An *in vitro *study with emphasis on biomarker discovery for atherosclerosis by proteomics, show elevated YKL-40 levels in the supernatant of macrophages following treatment with oxidized low-density lipoprotein, a process that mimics the formation of "foam cells" [44]. This also suggests a role of YKL-40 in the differentiation of monocytes to lipid-laden macrophages during formation of the atherosclerotic plaque.

### YKL-40 in cardiovascular disease

In the last few years, several clinical studies have described elevated YKL-40 levels in several cardiovascular conditions as well as described an association between YKL-40 and mortality. Studies show, that elevated YKL-levels are independently associated with the presence of CAD [35,36,45]. One study even found, that YKL-40 levels increase with the extent of CAD defined by the number of stenosed vessels as assessed by coronary angiography [35]. This findings indicate, that plasma YKL-40 levels could be a quantitative indicator of disease progression as well as of disease presence [35].

In patients suffering myocardial infarction (MI) even higher YKL-levels have been documented [36,45,46] and YKL-levels remain higher in patients with prior MI compaired to individuals without previous MI [45]. There seems to be no difference in YKL-40 levels between MI patients with or without ST elevations, but higher YKL-40 levels were seen in thrombolyzed patients compared with non-thrombolyzed patients during the first 24 hours after the event [46] indicating that YKL-40 is released from the dissolved thrombosis. Lately, elevated YKL-40 levels have also been documented in individuals with atrial fibrillation (AF) where the highest YKL-40 levels were found in patients with permanent AF compared to patients with persistent AF suggesting an association between the chronicity of AF and the inflammatory burden [34].

**Figure 1:**
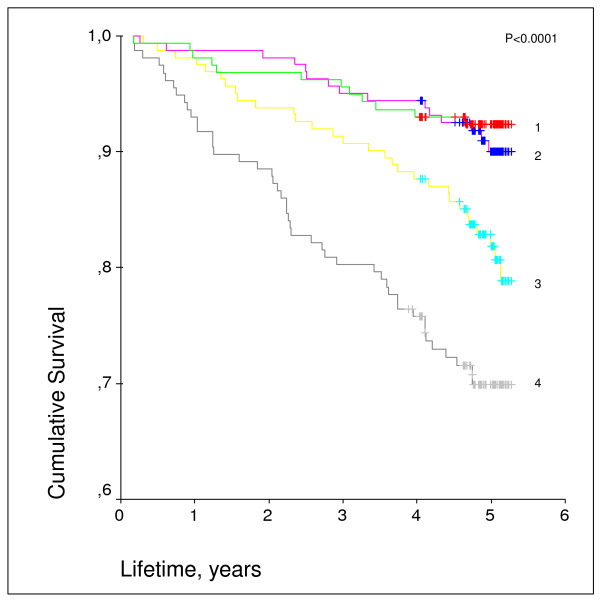
**Kaplan-Meier-curves of the unadjusted cumulative overall survival according to increasing quartiles of YKL-40 at baseline (59.5 ng/ml)**. Curves presented from top: The mortality rate was 1) 7.6% for first quartile values of YKL-40 (≤ 39 ng/l); 2) 9.3% for second quartile values of YKL-40 (39.1-59.5 ng/l); 3) 18.5 % for third quartile values of YKL-40 (59.6-111 ng/l), and 4) 29.3 % for four quartile values of YK-40 (> 111 ng/l), p < 0.0001. Y-axis represents the lifetime of the participants during the 5 years follow-up period.

Elevated YKL-40 levels have also been found to be associated with all-cause as well as cardiovascular mortality in patients with stable CAD [45]. Furthermore, increasing mortality rates with increasing YKL-40 levels at baseline are also seen over a 5 year period in the general population above 50 years of age without known diabetes or CVD (Figure 1) in which YKL-40 were also found to be an independent predictor of overall as well as of cardiovascular mortality (Table 1) [32].

**Table 1:** Hazard ratios for cardiovascular and overall mortality at 5 years follow-up in accordance to baseline values of YKL-40 in a representative group of the general population without known cardiovascular disease or diabetes

		HR (95% CI)		
	Cardiovascular mortality	P value	Overall mortality	p value
Unadjusted	2.16 (1.67-2.80)	< 0.0001	2.29 (1.53-3.44)	< 0.0001
Age- and sex-adjusted	1.75 (1.29-2.37)	< 0.0001	1.99 (1.26-3.16)	0.003
Mutivariable model	1.57 (1.16-2.14)	0.004	1.57 (1.00-2.46)	0.049

1 SD increase in ln variable.

N = 482, cardiovascular mortality events = 22, overall mortality events = 45.

* Variables: hypertension, total cholesterol, smoking, hsCRP, NT-proBNP and UACR.

Abbreviations: CI, confidence interval; HR, hazard ratio; hsCRP, high sensitive C-reactive protein; NT-proBNP, N-amino terminal fragment of the prohormone brain natriuretic peptide; UACR, urinary albumin/creatinine ratio.

All together these findings suggest YKL-40 as a possible screening modality/diagnostic marker for progressing coronary atherosclerosis. It seems reasonable to speculate that serum YKL-40 could be used for monitoring the efficiency and sufficiency of medical treatment of patients with CAD and thereby assist the clinician in reducing the high occurrence of fatal cardiovascular events.

### YKL-40 and diabetes

Individuals with diabetes have in general a 2- to 4-fold increased risk of subsequent CVD [47]. Persistent microalbuminuria is associated with an increased risk of CVD in both patients with type 1 and type 2 diabetes [48-50]. Patients with type 1 diabetes have up to a 9-fold increased mortality risk from ischemic heart disease, excessively higher in patients under 30 years of age [51].

It has been demonstrated, that patients with type 1 diabetes as well as patients with type 2 diabetes have elevated plasma YKL-40 levels [31-33,37,52]. In type 2 diabetes patients YKL-40 levels are correlated with insulin resistance [31,37] and in a single study also with the diabetic lipid profile [31]. Some studies have also shown a correlation between YKL-40 and glycemic parameters such as hemoglobin A1c [52] and fasting glucose [37] whereas others have not [31,33].

**Figure 2:**
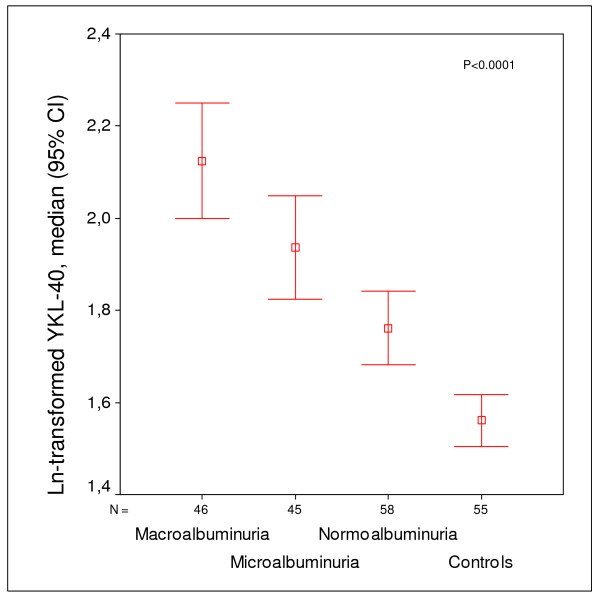
**Mean (95% confidence intervals) of ln-transformed YKL-40**. Equivalent YKL-40 data (median (interquartile range)), are for macroalbuminuria/diabetic nephropathy (U-albumin > 300 mg/24 h), YKL-40 = 117 (68-215) ng/ml; persistent microalbuminuria (U-albumin 30-300 mg/24 h), YKL-40 = 74 (45-160) ng/ml; normoalbuminuria (U-albumin < 30 mg/24 h), YKL-40 = 53 (32-105) ng/ml; control group, YKL-40 = 37 (29-52) ng/ml. P < 0.001 for all comparisons. Groups were matched according to gender and duration of diabetes (> 30 years).

In patients with type 1 diabetes a positive association between elevated plasma YKL-40 levels and increasing levels of albuminuria has been described (Figure 2) [52]. This finding indicates a role of YKL-40 in the progressing vascular damage in the kidneys resulting in complicating microvascular disease. This hypothesis is supported by the finding that YKL-40 and urinary albumin/creatinine ratio (UACR) are independent markers with only weak intercorrelation that seem to predict overall as well as cardiovascular mortality in a synergistic way in the general population above 50 years of age without known diabetes or CVD over a 5 year period (Table 2) [32].

**Table 2:** Unadjusted cumulative cardiovascular and overall mortality according to YKL-40 levels and UACR above and below median at baseline in a representative group of the general population without diabetes, hypertension or CVD

	Cardiovascular mortality*, %	Overall mortality*, %
YKL-40 < median, UACR ≤ median	0.7	4.4
YKL-40 > median, UACR ≤ median	1.1	8.0
YKL-40 ≤ median, UACR > median	3.0	7.6
YKL-40 > median, UACR > median	10.6	30.6

*p < 0.0001 for all comparisons.

N = 389, cardiovascular mortality events = 13, overall mortality events = 44.

Median value of YKL-40, 59.5 ng/ml, median value of UACR, 7 mg/g.

Abbreviation: UACR, urinany albumin/creatinine ratio.

A study of polymorphisms of the *CHI3L1 *locus encoding the inflammatory protein YKL-40 did not show any association between certain gene polymorphisms and the risk of type 2 diabetes. It therefore seems reasonable, that it is the low grade inflammation and endothelial dysfunction progressing to later micro- and macrovascular complications that account for the elevated YKL-40 levels in diabetic patients.

### YKL-40 in other clinical conditions

Serum YKL-40 levels have been found to be elevated in other clinical conditions not directly related to atherosclerosis or cardiovascular disease. Several studies describe elevated YKL-40 levels in patients with different types of cancer [9,11,53]. YKL-40 levels seem to be related to tumor grade and burden, short recurrence-free interval and short disease-free and overall survival [11]. The exact biological function of YKL-40 in cancer is unknown, but YKL-40 seems to play an important role in tumor invasion. The signalling pathways MAPK/ERK1/2 and PI3K/AKT which YKL-40 has been demonstrated to mediate its effects through in other conditions [26,28] are critical in the malignant phenotype of glioblastoma and have been shown to govern proliferation and survival, invasiveness and radiation resistance [54]. Furthermore, activation of the PI3K/AKT-pathway is correlated with increased tumor grade, lesser likelihood of apoptosis and decreased overall survival [54]. However, the functional ligand for the chitin-binding site in YKL-40 in relation to cancer is not presently known.

Recently, an *in vitro *study has shown, that ectopic expression of YKL-40 in breast and colon cancer cells respectively led to tumor formation with an extensive angiogenic phenotype and that recombinant YKL-40 protein promoted vascular endothelial cell angiogenesis whereas blockade of YKL-40 suppressed tumor angiogenesis both *in vitro *and *in vivo *[55]. Furthermore, immunohistochemical analysis of human breast cancer showed a correlation between YKL-40 expression and blood vessel density [55]. Therefore, the occurrence of high YKL-40 levels in highly differentiated and advanced cancers and recurrent cancer states could be explained by the role of YKL-40 in both angiogenesis and fibrogenesis, since highly differentiated tumours are characterized by high vascularization and a high turnover of extracellular matrix.

YKL-40 is not tumor specific and the studies of YKL-40 as a screening marker for cancer and as a marker useful for monitoring therapeutic results differ [9]. Furthermore, YKL-40 seems not suited as a tumor marker due to low specificity and sensitivity [9].

## Conclusion

Substantial evidence supports a role of YKL-40 in endothelial dysfunction, atherosclerosis and manifest CVD. Clinical studies have demonstrated, that YKL-40 levels are associated with the presence and extent of CAD, are even higher in patients with MI and are associated with all-cause as well as cardiovascular mortality. YKL-40 plays a role in relation to cell migration, reorganization and tissue remodelling during atherogenesis and seems to play a pivotal role in the differentiation of monocytes to activated macrophages in tissues characterized by inflammation. However, the YKL-40 receptor(s) still remain to be isolated and described.

YKL-40 has emerged as a promising marker of cardiovascular disease. It seems to be useful for screening because it is detectable in early stage subclinical disease, and it also seems to have the potential of becoming a prognosticator of cardiovascular events and mortality. Future research around YKL-40 should concentrate further on establishing whether YKL-40 could assess the value of a cardiovascular biomarker in clinical practice. Therefore, further investigations of YKL-40 in relation to CAD, MI and diabetes are needed as well as intervention studies describing possible changes in serum/plasma YKL concentrations concomitant with optimized medical treatment of conditions such as e.g. angina pectoris and diabetes. Furthermore, to assess the value as a useful marker in clinical practice, both specificity and sensitivity of YKL-40 in relation to CVD need to be clarified and optimized.

Studies in obese with and without complications such as CVD and/or diabetes are few. Such studies could also contribute in establishing YKL-40 as a useful cardiovascular biomarker, since the weight loss following bariatric surgery is accompanied by a reduced risk of CVD in these patients. One study has described significantly reduced YKL-40 levels in obese having bariatric surgery indicating an association between serum YKL-40 levels and adipose tissue/weight loss/reduced cardiovascular risk that still remains to be clarified [33]. Cardiovascular follow-up in these patients should be done.

Finally, elevated YKL-40 levels have also been observed in patients with highly differentiated and advanced cancers of various types as well as recurrent cancer states, but recent studies show that this could be explained by the role of YKL-40 in cancer angiogenesis and fibrogenesis.

## Competing interests

The authors declare that they have no competing interests.

## Authors' contributions

CNR drafted and finished the manuscript. HV made critical revision of the manuscript. Both authors have read and approved the final manuscript.

## Authors' information

CNR is one of the leading scientists worldwide in the field of YKL-40 in releation to diabetes, atherosclerosis and cardiovascular disease. CNR was the first to describe elevated YKL-40 levels in patients with type 2 diabetes and is the only scientist who has examined YKL-40 levels in patients with type 1 diabetes. Beside clinical studies in patients with diabetes and/or cardiovascular disease, CNR conduct cellular studies which hopefully will elucidate the mechanisms by which YKL-40 mediates its function. CNR obtain a Ph.D on this research field in january 2010.

HV is chief scientist with primary focus on diabetes and micro- and macrovascular complications. HV is supervisor of CNR and has made substantial contributions to concept and design of the YKL-40 studies conducted by the research group.
